# A Patient Develops Bullous Rash After Receiving the Second Dose of COVID-19 Vaccine

**DOI:** 10.7759/cureus.29786

**Published:** 2022-09-30

**Authors:** Syeda S Nida, Gabriel J Tobon, Morgan Wilson, Krati Chauhan

**Affiliations:** 1 Internal Medicine, Southern Illinois University School of Medicine, Springfield, USA; 2 Internal Medicine, Rheumatology, Southern Illinois University School of Medicine, Springfield, USA; 3 Internal Medicine, Dermatology, Southern Illinois University School of Medicine, Springfield, USA

**Keywords:** covid-19 vaccine complication, vaccine related rash, autoimmune blistering skin disease, pfizer vaccine, bullous pemphioid

## Abstract

Our knowledge about the clinical spectrum of COVID-19 has continued to evolve. The clinical features of the infection and vaccine are continuously updated. We present a case of bullous pemphigoid after receiving a second dose of the COVID-19 vaccine. This case highlights autoimmune skin findings seen in a patient after COVID-19 vaccination.

A 70-year-old male presented with the chief complaint of blistering skin rash. He received his second dose of Pfizer COVID-19 vaccine two days before developing a painful pruritic maculopapular rash that started on his hands and extended proximally to his trunk. Physical exam was remarkable for tense bullae with negative Nikolsky sign. Biopsy and direct immunofluorescence lead to the diagnosis of bullous pemphigoid. The lesions improved significantly with steroids.

Various cutaneous eruptions have been reported with Moderna and Pfizer COVID-19 vaccines, including the new onset of bullous pemphigoid. Based on our case, we suggest that bullous pemphigoid after COVID-19 vaccination is responsive to steroids and the prognosis is excellent. Understanding the clinical course and prognosis of bullous pemphigoid from the COVID-19 vaccine is of significant importance as we strive to keep our patients and communities safe. More data is needed to better guide recommendations, but so far looking at the example from our case, the benefits of COVID-19 vaccination seem to outweigh the risks. Therefore, patients should be advised to continue with future vaccinations.

## Introduction

Bullous pemphigoid is an autoimmune disease that is characterized by fluid-filled subepithelial blister formation that affects the skin and mucosal membranes. The condition can have many triggering or associating factors, and in recent years, there has been an increasing number of bullous pemphigoid cases post-COVID-19 mRNA vaccination. In this case report, we present a patient who developed a bullous rash after receiving the second dose of the COVID-19 mRNA vaccine which was later proven as bullous pemphigoid through biopsy.

## Case presentation

A 70-year-old male with a past medical history significant for Parkinson’s disease, coronary artery disease, stage III chronic kidney disease, obstructive sleep apnea, chronic anemia, alcohol abuse disorder, renal cell carcinoma status post right radical nephrectomy, and squamous cell carcinoma of the lung in remission after chemoradiation, presented with the chief complaint of blistering skin rash.

The patient received his second dose of Pfizer COVID-19 vaccine two days before developing a painful pruritic maculopapular rash that started on his hands and extended proximally to his trunk. He was seen in the emergency department (ED) and discharged home on permethrin for presumed scabies. Due to a lack of improvement, he came back to the ED after one week. At this time, the rash had evolved into tense bullae and involved most of his body without any mucosal involvement. This development warranted hospital admission. He denied fever, chills, dyspnea, cough, nausea, or vomiting. No sick contacts were reported.

**Figure 1:**
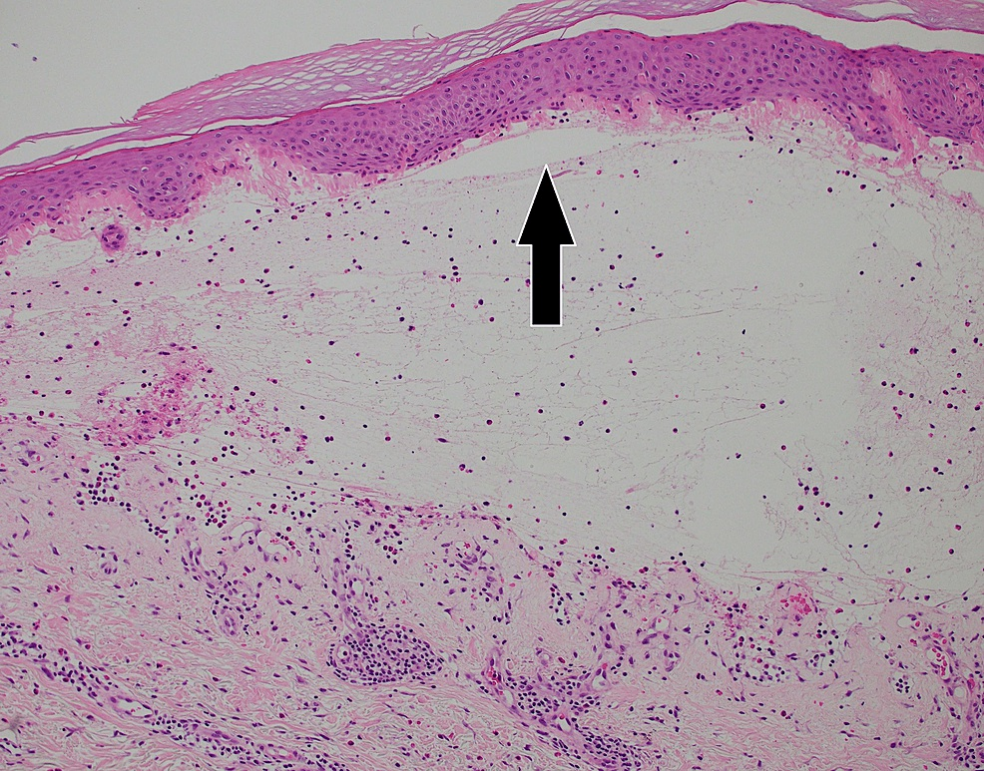
Subepidermal bulla (H/E stain, original magnification x100) H/E: Haemotoxylin and eosin Image courtesy of Dr. Wilson Morgan and the Dermatopathology Service at the Southern Illinois University School of Medicine

**Figure 2:**
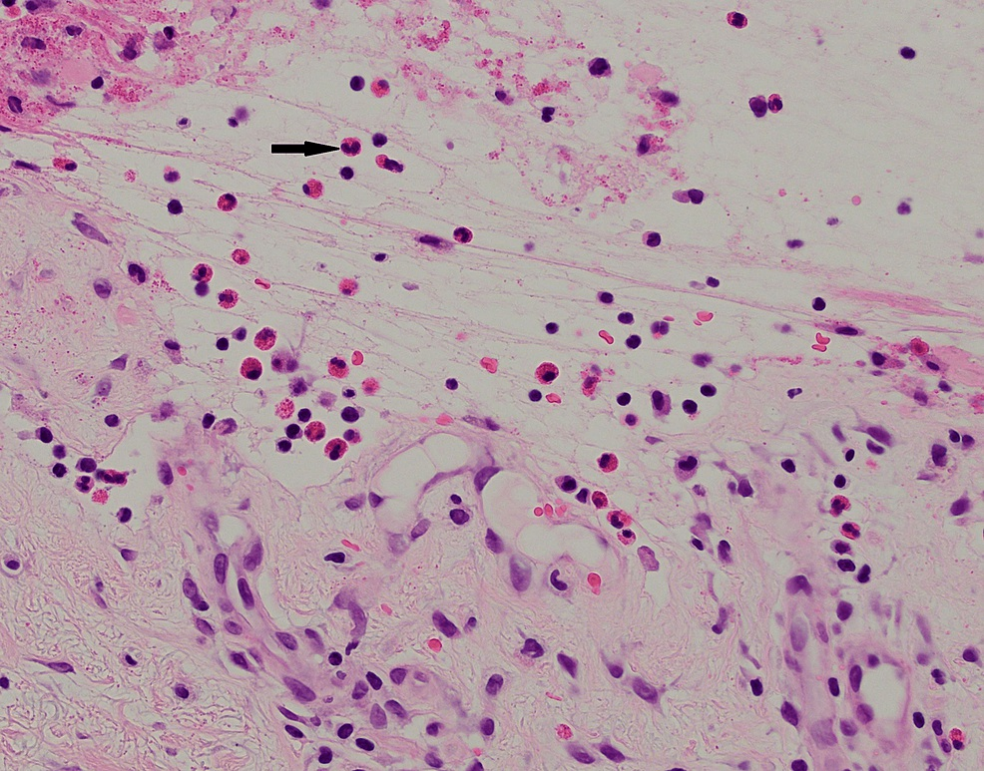
Eosinophils within the bulla cavity, a common finding in bullous pemphigoid. H/E: Haemotoxylin and eosin Image courtesy of Dr. Wilson Morgan and the Dermatopathology Service at the Southern Illinois University School of Medicine

**Figure 3:**
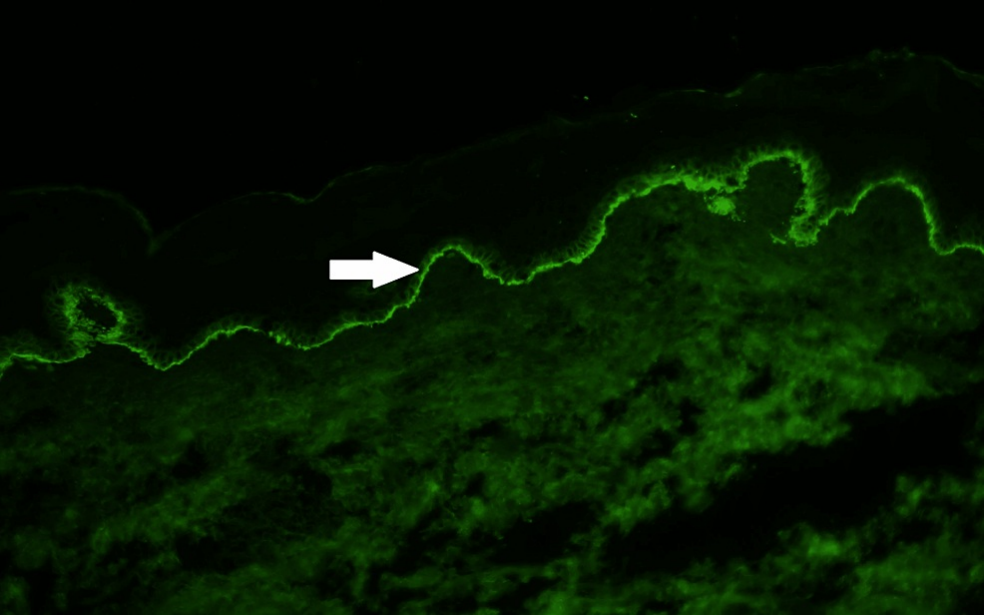
Direct immunofluorescence biopsy showing weak linear deposition of IgG and strong linear deposition of C3 along the basement membrane zone (white arrow). IgG: Immunoglobulin G, C3: Complement 3 Image courtesy of Dr. Wilson Morgan and the Dermatopathology Service at the Southern Illinois University School of Medicine

The etiology of the rash was initially thought to be drug-related since he was recently started on a new medication, pimavanserin, for Parkinson’s disease treatment. Despite discontinuing the medication for more than one week, his rash continued to progress, warranting further investigation. The patient did not receive any recent chemoradiation or immunotherapy. His last radiation therapy was approximately two years ago. Physical exam was remarkable for tense bullae with negative Nikolsky sign. Laboratory work was remarkable for elevated erythrocyte sedimentation rate at 40 mm/hour, mildly elevated absolute eosinophils count at 0.6 k/cumm, and normal complement 3 (C3) level at 0.8 g/dl. Punch biopsy showed a sub-epidermal bulla with eosinophils (Figures 1-2). Direct immunofluorescence showed weak linear deposition of immunoglobulin G (IgG) and strong linear C3 deposition at the dermal-epidermal junction (Figure 3). The combination of morphological pattern, direct immunofluorescence, and expert opinion from the dermatology team lead to a diagnosis of bullous pemphigoid. After the initiation of topical and systemic steroids (prednisone and clobetasol), the lesions improved significantly. His hospital course remained uneventful and he was discharged to a rehabilitation facility. He was discharged on prednisone 60 mg daily. The patient was recommended to continue prednisone 60 mg for a total of one week, with full skin examinations every day to ensure no new blisters. After one week, prednisone was decreased to 40mg daily for one week, then 30mg daily for one week, then 20mg daily, then 10mg daily until his follow-up appointment with dermatology. He was also discharged on topical clobetasol propionate 0.05% ointment for a total of two weeks.

## Discussion

Bullous pemphigoid (BP) is an autoimmune blistering disease characterized by subepithelial blister formation and deposition of immunoglobulins and complements within the epidermal basement membrane toward hemidesmosomal antigens BP180 and BP230. Phenotypical features include tense, fluid-filled bullae of skin. Bullous pemphigoid can be idiopathic or triggered by several factors such as ultraviolet light, radiation, and medications. Vaccines that have been linked to BP include influenza, varicella, pneumococcus, meningococcus, tetanus, diphtheria, and hepatitis B. Various cutaneous eruptions have been reported with Moderna and Pfizer COVID-19 vaccines, including the new onset of bullous pemphigoid [1-6]. 

Calabria et al. reported 35 severe autoimmune blistering disease cases triggered by the SARS-CoV-2 vaccination. Of which, 26 (74.3%) were diagnosed with BP, two (5.7%) with linear immunoglobulin A (IgA) bullous dermatosis, six (17.1%) with pemphigus vulgaris, and 1 (2.9%) as pemphigus foliaceus. The mean age of the sample was 72.8 years and there was a predominance of males over females (F:M=1:1.7). In 22 (62.9%) cases, the disease developed after Pfizer vaccine administration, 6 (17.1%) after Moderna, 3 (8.6%) after AstraZeneca, 3 (8.6%) after CoronaVac, and one was not specified [7].

Maronese et al. reported new onset and reactivation of BP in 21 patients after receiving severe acute respiratory syndrome coronavirus 2 (SARS-CoV-2) vaccines. Seventeen patients received the Pfizer vaccine, two received the Moderna mRNA-1273 vaccine, one received the AstraZeneca vaccine, and another received the first dose of the AstraZeneca vaccine and the second dose of the Pfizer vaccine. The median latency between vaccine administration and the onset of cutaneous eruptions was 27 days and the sample size was too small to accurately associate with a specific vaccine. The mean age of the patients was 79 years and there was a predominance of males over females. The onset of disease was reported both after the first and second dose with a similar latency period, however, recurrence of disease was uncommon [8]. Our patient received his booster dose eight months later and had an uneventful post-vaccination course. All patients were treated with topical and/or systemic corticosteroids, with or without the addition of immunosuppressive drugs, with a good clinical response in every case [8]. 

## Conclusions

Based on the review of our case and other similar cases, we find that the clinical course of BP after COVID-19 vaccination is usually benign and the prognosis is excellent. Understanding the clinical course and prognosis of BP from the COVID-19 vaccine is of significant importance as we strive to keep our patients and communities safer with booster doses. More data is needed to better guide recommendations, but going by the example from our case and other similar cases, the benefits of COVID-19 vaccination seem to outweigh the risks. And therefore, patients should be advised to continue with future vaccinations.
